# Identification prognosis-associated immune genes in colon adenocarcinoma

**DOI:** 10.1042/BSR20201734

**Published:** 2020-11-17

**Authors:** Yandong Miao, Jiangtao Wang, Xueping Ma, Yuan Yang, Denghai Mi

**Affiliations:** 1 The First Clinical Medical College of Lanzhou University, Lanzhou City, Gansu Province, P.R. China; 2 Second People’s Hospital of Gansu Province, Lanzhou City, Gansu Province, P.R. China; 3 Gansu Academy of Traditional Chinese Medicine, Lanzhou City, Gansu Province, P.R. China

**Keywords:** Bioinformatic analysis, Colon adenocarcinoma, Immune cell infiltration, Immune gene, TCGA, tumor microenvironments

## Abstract

Colon adenocarcinoma (COAD) is one of the most prevalent malignant tumors worldwide. Immune genes (IGs) have a considerable correlation with tumor initiation and prognosis. The present paper aims to identify the prognosis value of IGs in COAD and conduct a prognosis model for clinical utility. Gene expression data of COAD were downloaded from The Cancer Genome Atlas (TCGA), screening and analyzing differentially expressed IGs by bioinformatics. Core genes were screened by univariate and multivariate Cox regression analyses. Survival analysis was appraised by the Kaplan–Meier method and the log-rank test. Gene Ontology, Kyoto Encyclopedia of Genes and Genomes, and Gene Set Enrichment Analysis (GSEA) were used to identify IGs’ relevant signal pathways. We predicted the overall survival (OS) by nomogram. Finally, a prognosis model was conducted based on 12 IGs (*SLC10A2, CXCL3, NOX4, FABP4, ADIPOQ, IGKV1-33, IGLV6-57, INHBA, UCN, VIP, NGFR*, and *TRDC*). The risk score was an independent prognostic factor, and a nomogram could accurately predict the OS of individual COAD patients. These results were validated in GSE39582, GSE12945, and GSE103479 cohorts. Functional enrichment analysis demonstrated that these IGs are mainly enriched in hormone secretion, hormone transport, lipid transport, cytokine–cytokine receptor interaction, and peroxisome proliferators-activated receptor signaling pathway. In summary, the risk score is an independent prognostic biomarker. We also excavated several IGs related to COAD’s survival and maybe potential biomarkers for COAD diagnosis and treatment.

## Introduction

Colorectal cancer is the fourth of the new cases and deaths among 36 cancers in 2018 [1]. It is also the second most frequent cancer diagnosed in women and the third most in men [2]. Several clinical trials have shown that the enhancement of tumor immune response can lead to long-term clinical response and benefit. Many expression profiling studies have proved that there is a relationship between the immune genes’ (IGs) characteristics and the better prognosis or efficacy to therapy, either chemotherapy or immunotherapy in tumors [3,4]. It is also essential to understand the immune environment’s contribution and the molecular subtypes of different tumor indications, but it lacks adequate wisdom to promote a reasonable combination of immunotherapy [5,6].

Whole genome-wide gene expression clusters such as The Cancer Genome Atlas (TCGA) have been constructed to classify and identify genomic abnormalities in large populations worldwide to detect the effects of the genetic composition of tumors on clinical prognosis [7]. With the human genome map finished, gene expression profiling has been cumulatively accepted by clinical diagnostic criteria. Besides, tumor microenvironment (TME) has significantly influenced therapeutic response and clinical outcomes [8,9]. Transcription and genomic data stemmed from many tumor samples used to research the TME, and immune infiltration assessment illustrated molecular subtypes of acute myeloid leukemia, bladder cancer, and cervical cancer [10–12]. Several separate reports about immune infiltration and lncRNA were associated with colon adenocarcinoma (COAD) [13,14]. Immunoscore, the primary significant prognostic ability, and robustness in COAD were validated [15]. However, few studies on the combination of IG and immune infiltration in the prognosis model of COAD patients.

The present paper mainly attempted to construct the prognosis model based on prognosis-related IGs (PRIGs), explore the prognostic value, and inquiry immune infiltration in COAD, which provide a novel orientation to the potential targets. Besides, we validated the PRIGs signature in GSE39582, GSE12945, and GSE103479 cohorts. In summary, we identified a new essential marker (risk score) to predict COAD patients’ prognosis. Comparison with the previous research, our study provided a new risk model as an independent prognostic biomarker in risk stratification for COAD patients.

## Materials and methods

### Data sources

In the present study, both the transcriptome and clinical data of COAD were obtained from the TCGA database (Data Release 20.0, Release Date: 11 November 2019, https://tcga-data.nci.nih.gov/tcga/). The main filter criteria for our data were as follows: (1) The keywords for cases were colon [Primary Site], ‘TCGA [Program],’ ‘TCGA-COAD [Project],’ ‘Adenomas and Adenocarcinomas [Disease Type].’ (2) The keywords for files for RNA‐sequencing data were ‘Transcriptome Profiling [Data Category],’ ‘Gene Expression Quantification [Data Category],’ ‘RNA-Seq [Experimental Strategy],’ ‘HTSeq - FPKM [Workflow Type]’). (3) The keywords for files for clinical data were ‘Clinical [Data Category],’ ‘BCR XML [Data Format]’). The expression of mRNA was obtained from the RNA‐sequencing data. The data access (GSE39582, GSE12945, and GSE103479), as the validation data, was obtained from Gene Expression Omnibus (GEO, https://www.ncbi.nlm.nih.gov/geo/) database. Gene chips’ raw data were normalized using the Robust Multi-Array Average (RMA) algorithm provided by ‘limma’ [16]. Perl and R-package ‘sva’ were used to merge microarray data and reduce heterogeneity among the GSE39582, GSE12945, and GSE103479 datasets [17]. We use R software (version 3.6.2) to extract and annotate the expression of immune genes in TCGA and GEO databases.

### Bioinformatics analysis of the data

#### Identification of differential expression of genes, IGs, and transcription factors

Data extraction and integration were performed by Perl software. Screening differentially expressed genes, IGs, transcription factors (TFs) was undertaken by the Wilcoxon Test [16], using R-package ‘limma’ ‘edgeR.’False-discovery rate (FDR) < 0.05 and Log2 | (fold change, FC) | > 1.5 were set as the cutoffs. Bidirectional hierarchical clustering was analyzed and heat map was drawn by R-package ‘pheatmap’ (https://cran.r-project.org/web/packages/pheatmap/). R-package ‘ggplot2’ was used to draw a volcano map. Correlation analysis between differentially expressed IGs (DEIGs) and TFs (DETFs) was performed using correlation tests based on R software (cor > 0.4, *P*<0.001). The protein–protein interaction (PPI) network was constructed by Cytoscape software (version 3.7.2) [18]. R-package ‘Survival’ was used to conduct univariate cox regression analysis (*P*<0.01). Forest map was drawn by the R language ‘ggforest function.’

#### Building a prognosis model of IGs and survival analysis

We constructed an optimal prognosis model of PRIGs via the Akaike Information Criterion. Genes expression and survival analysis were evaluated by the Kaplan–Meier method and the log-rank test (*P*<0.05). The risk score of each patient was calculated by the following formula: $\text{Risk}\;\text{score}=\Sigma_{j=1}^{\text{n}}\;Coefj\;\times Xj$, where, Coefj denotes the coefficient and Xj indicates the expression levels of each IG [19]. The survival data were obtained from the clinical data and combined with the previously acquired expression profiling data. The median risk score was selected as a cutoff to divide COAD cohorts into high-risk and low-risk groups. The evaluation indicator of survival analysis was overall survival (OS), which refers to the period from the date of diagnosis until death from any cause. The survival curve was drawn according to the high- and low-risk value using R-package ‘survival’ and ‘survminer.’ R-package ‘survival ROC’ was used to draw the Receiver Operating Characteristic (ROC) curve for the difference and calculate the value of Area Under Curve (AUC). The risk curve, survival state diagram, and heat map were drawn based on the patient risk score. PRIGs were recognized by univariate and multivariate Cox regression analyses. The results were verified in the GSE39582, GSE12945, and GSE103479 cohorts.

#### The exploitation of the nomogram

We used the expression of PRIGs to draw a nomogram via the R-package ‘Hmisc,’ ‘lattice,’ ‘Formula,’ ‘ggplot2,’ ‘foreign,’ and ‘rms.’ Moreover, calibration curves were used to assess the consistency between actual and predicted survival rates. Furthermore, the consistency index (C-index) was calculated to evaluate the model prognosis capability. The values of 0.5 and 1.0 represent a random probability and an excellent performance for predicting survival with the model, respectively. The GSE39582, GSE12945, and GSE103479 cohorts were used to verify the risk score’s prognostic value and the nomogram.

#### Functional enrichment analysis of PRIGs and immune cells infiltration in COAD

Based on the R software, the functional enrichment analysis of PRIGs was performed to identify Gene Ontology (GO) categories of biological processes (BPs) and molecular functions (MFs). The Database for Annotation, Visualization and Integrated Discovery (DAVID) database was used to conduct enrichment analysis of Kyoto Encyclopedia of Genes and Genomes (KEGG) pathways [20]. FDR < 0.05 was invoked as the cutoff. R-package ‘GOplot’ was used to integrate expression data and functional enrichment analysis. Moreover, Gene Set Enrichment Analysis (GSEA) was also used to analyze the differences between the high-risk and low-risk groups. The number of permutations was set to 1000 and FDR < 0.25 was acknowledged as statistically significant [21].

## Results

### Characteristics of patients

TCGA-COAD cohort included 385 patients. Patients without survival time and less than 90 days were excluded, and 334 patients were obtained as the training group. GSE39582 dataset included 566 stage I–IV COAD patients, GSE12945 contained 62 stages I–IV colorectal cancer patients, and GSE103479 included 156 stage I–IV colorectal cancer patients. Using the same exclusion criteria of the training group, 587 colorectal cancer patients were selected from the GEO cohorts as the test group. The detailed clinical characteristics of patients are listed in Supplementary Table S1.

### Identification of differentially expressed genes, IGs, and TFs

**Figure 1:**
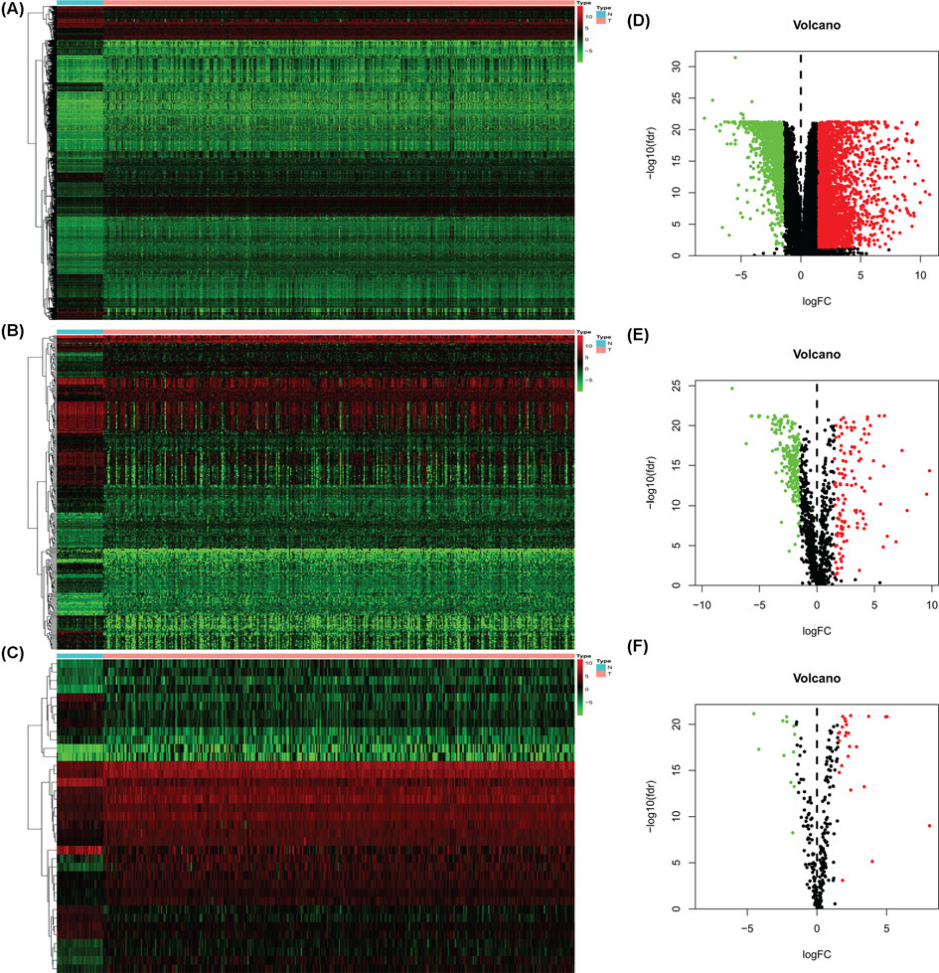
Differentially expressed genes, IGs, and TFs in TCGA-COAD Heatmaps: DEGs (**A**), DEIGs (**B**), and DETFs (**C**); higher expression manifested in red; lower expressions were green. Volcano diagrams: DEGs (**D**), DEIGs (**E**), and DETFs (**F**); each point represents a gene. Down-regulated (green), up-regulated (red), and no significance DEGs, DIEGs, DTFs (black). IGs, immune genes; TFs, transcription factors;TCGA, The Cancer Genome Atlas; COAD,colon adenocarcinoma; DEGs, differentially expressed genes;DEIGs, differentially expressed immune genes; DETFs, differentially expressed transcription factors.

A total of 437 transcriptome data were obtained, 39 (8.9%) were derived from healthy samples and 398 cases (91.1%) came from tumor samples. We screened out 3833 differentially expressed genes (DEGs) included 2610 up-regulated and 1223 down-regulated (Figure 1A,D); 332 differentially expressed immune genes (DEIGs) contained 120 up-regulated and 212 down-regulated (Figure 1B,E). We also obtained 37 differentially expressed transcription factors (DETFs) including 25 up-regulated and 12 down-regulated (Figure 1C,F).

### Prognosis model of immune constructed and survival analysis

We filtered out 24 PRIGs by univariate Cox regression analysis. CXCL3 was low-risk PRIG (Hazard Ratio (HR) < 1, *P*<0.01), others were high-risk PRIGs (HR > 1, *P*<0.01); the detailed information is shown in Supplementary Figure S1 and Table S2. Twelve PRIGs were selected by multivariate regression analysis, including *SLC10A2, CXCL3, NOX4, FABP4, ADIPOQ, IGKV1-33, IGLV6-57, INHBA, UCN, VIP, NGFR*, and *TRDC* (Table 1). The prognosis model was constructed based on these 12 PRIGs. The coefficient of PRIGs is shown in Supplementary Figure S2. Each patient’s risk score was computed based on each gene’s expression level and risk coefficient. A risk score was used to predict prognosis.

**Table 1:** PRIGs (multivariate Cox regression analysis) and prognosis model coefficient

PRIGs	Coefficient	HR	95% CI	*P*-value
SLC10A2	0.6105	1.8413	1.1402−2.9734	0.0125
CXCL3	−0.0249	0.9754	0.9567−0.9945	0.0119
NOX4	−1.1528	0.3157	0.0985−1.0122	0.0524
FABP4	0.0693	1.0717	1.0286−1.1167	0.0009
ADIPOQ	−0.3076	0.7352	0.5597−0.9657	0.0270
IGKV1-33	0.0626	1.0646	1.0186−1.1126	0.0054
IGLV6-57	0.0027	1.0027	1.0012−1.0042	0.0004
INHBA	0.1227	1.1306	1.0327−1.2377	0.0079
UCN	0.4528	1.5727	1.2582−1.9658	0.0001
VIP	0.0473	1.0485	0.9867−1.1141	0.1266
NGFR	−0.3930	0.6750	0.4345−1.0489	0.0805
TRDC	0.1966	1.2173	1.0900−1.3595	0.0005

The calculation formula of the risk score was as follows: Risk score = 0.1966 × expression of TRDC + (−0.3930) × expression of NGFR + 0.0473 × expression of VIP + 0.4528 × expression of UCN + 0.1227 × expression of INHBA + 0.0027 × expression of IGLV6-57 + 0.0625 × expression of IGKV1-33 + (−0.3076) × expression of ADIPOQ + 0.0692 × expression of FABP4 + (−1.1528) × expression of NOX4 + (−0.0249) × expression of CXCL3 + 0.6105 × expression of SLC10A2.

**Figure 2:**
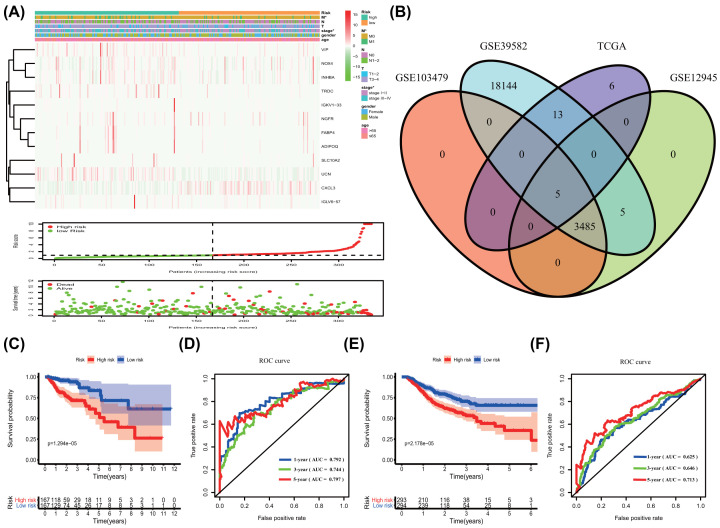
Prognosis model of IGs and survival analysis (**A**) Heatmap of the immune-associated gene expression profiles in prognostic signature for TCGA-COAD. The distribution of risk score and patient’s survival time, as well as the survival status of TCGA-COAD. The black dotted line is the optimum cutoff dividing patients into low- and high-risk groups. * represented *P*<0.05. (**B**–**F**) Kaplan–Meier survival analysis of COAD patients ranked by the median risk score. The high-risk score was related to poor OS in training group (C) and test group (E). ROC analysis of the sensitivity and specificity of the OS in training group (D) and test group (F). OS, overall survival; ROC, Receiver Operating Characteristic.

According to the median risk score, the patients were divided into high-risk and low-risk groups. A heat map visualized the gene expression profiles of the high- and low-risk groups. As the risk score escalated, the patients’ survival time decreased, and the death numbers increased (Figure 2A). In the training group, the Venn diagram and R software were used to select the validation gene by intersecting with the 24 prognostic-related IGs screened from the TCGA database, and finally 5 genes were verified (Figure 2B). OS was significantly lower in the high-risk subgroup than in the low-risk subgroup (Figure 2C, *P*=1.294e−05), the 1-, 3-, and 5-year AUC value of ROC was 0.792, 0.744, and 0.797, respectively (Figure 2D). Similarly, in the test group, OS was also apparently lower in the high-risk subgroup than in the low-risk subgroup (Figure 2E, *P*=2.178e−05), and the 1-, 3-, and 5-year AUC value of ROC was 0.625, 0.646, and 0.713, respectively (Figure 2F). Univariate and multivariate independent prognostic analyses manifested that the risk score was an independent prognostic predictor both in the training and test groups (Tables 2 and 3, Supplementary Figure S3).

**Table 2:** The prognostic value of different clinical characteristics in the training group

Variables	Univariate prognostic analysis			Multivariate prognostic analysis		
	HR	95% CI	*P*-value	HR	95% CI	*P*-value
**Age (≤65 vs. >65 years)**	1.7361	0.8958–3.3647	0.1023	2.4514	1.1910–5.0456	0.0149
**Gender (female vs. male)**	1.1784	0.6496–2.1375	0.5890	0.9428	0.5054–1.7587	0.8530
**Stage (I/II vs. III/IV)**	4.5987	2.3719–8.9160	6.28E-06	2.1689	0.2393–19.6625	0.4912
**T (1–2 vs. 3–4)**	9.5372	1.3085–69.5115	0.0261	3.8526	0.5048–29.4052	0.1933
**N (0 vs. 1–3)**	4.2744	2.2413–8.1519	1.03E-05	1.2869	0.1687–9.8185	0.8078
**M (0 vs. 1)**	6.6080	3.6126–12.0868	8.84E-10	3.4409	1.6652–7.1102	0.0008
**Risk score**	1.0069	1.0037–1.0101	2.46E-05	1.0051	1.0018–1.0083	0.0021

**Table 3:** The prognostic value of different clinical characteristics in the test group

Variables	Univariate prognostic analysis			Multivariate prognostic analysis		
	HR	95% CI	*P*-value	HR	95% CI	*P*-value
**Age (≤65 vs. >65 years)**	1.3467	0.9954**–**1.8221	0.0536	1.4041	1.0328–1.9087	0.0303
**Gender (female vs. male)**	1.2162	0.9046–1.6352	0.1950	1.2656	0.9367–1.7101	0.1250
**Stage (I/II vs. III/IV)**	2.0615	1.5306–2.7766	1.92E-06	2.2131	1.6259–3.0124	4.42E-07
**T (1–2 vs. 3–4)**	2.0090	1.0613–3.8027	0.0321	1.3989	0.7332–2.6689	0.3084
**N (0 vs. 1–3)**	0.9644	0.7186–1.2943	0.8091	0.7357	0.5394–1.0034	0.0526
**M (0 vs. 1)**	1.2951	0.8288–2.0235	0.2562	1.4461	0.9076–2.3042	0.1207
**Risk score**	1.7018	1.4854–1.9498	1.84E-14	1.6438	1.4254–1.8958	8.44E-12

### A characterized prognostic prediction model

**Figure 3:**
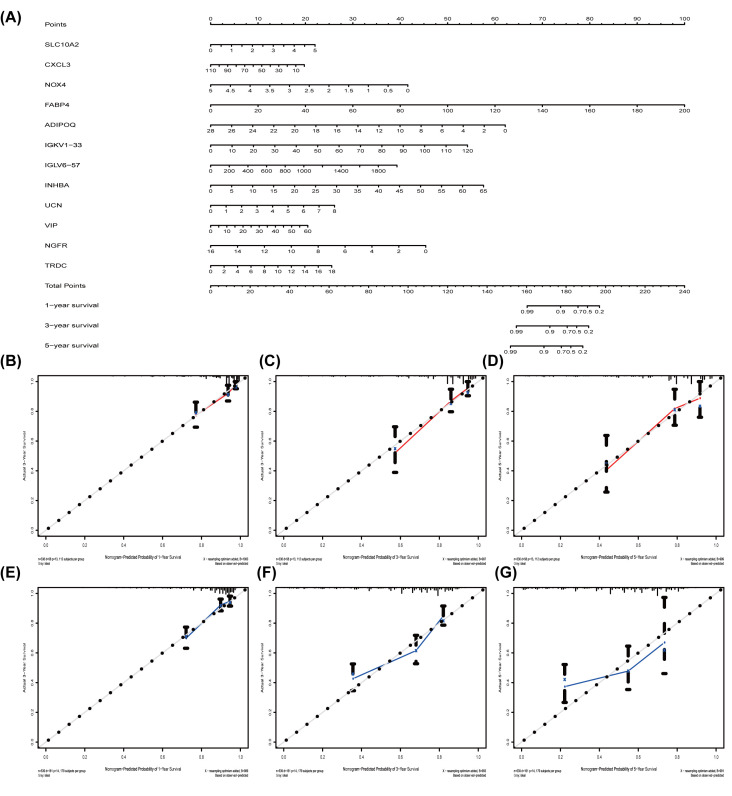
The nomogram to anticipate prognostic probabilities in COAD (**A**) The nomogram for predicting 1-, 3-, and 5-year OS of TCGA-COAD by 12 PRIGs signature. (**B–G**) The nomogram for predicting 1-, 3-, and 5-year OS of COAD by gene expression. (B–D) TCGA dataset and (E–G) GEO dataset. OS, overall survival.

Nomogram is a useful tool quantifying an individual’s risk in a clinical setting by integrating multiple risk factors [22,23]. We exploited the nomogram to predict 3- and 5-years OS probabilities by integrating the 12 PRIGs signature for TCGA-COAD and verified in the GEO datasets (Figure 3A). Each factor is assigned a score in proportion to its contribution to the survival risk. We calculated each patient’s total points by summarizing the number of points for all PRIGs and estimating survival rates via drafting a vertical line between each prognosis axis and the total point axis, which may help appropriate practitioners make clinical decisions for COAD patients. The calibration curve showed that the actual survival rate matched the predicted 3- and 5-years survival rate well; the C-index was 0.77 in the TCGA data (Figure 3B–D). The nomogram was verified in the GEO cohorts, the C-index was 0.72, and 3- and 5- year calibration curves were shown in Figure 3E–G.

### Functional enrichment analysis of PRIGs and immune cells infiltration in TCGA-COAD

**Figure 4:**
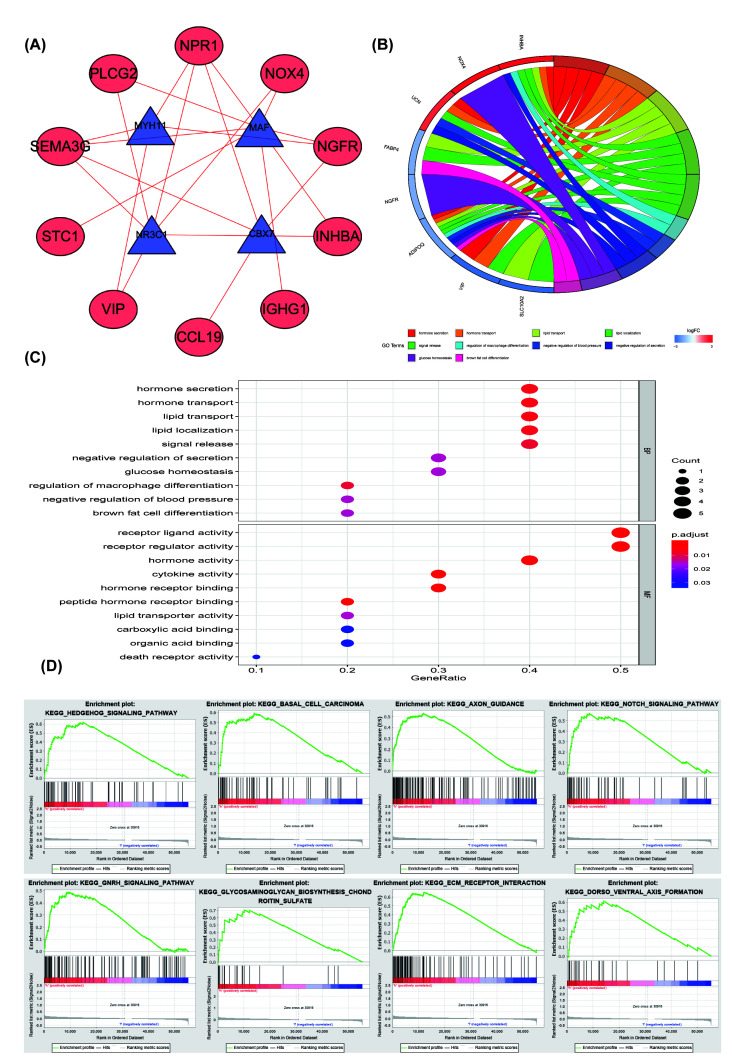
PPI networks and functional enrichment analysis of PRIGs (**A**) PPI networks: the positive interactions (red line), TFs (blue and triangle), high-risk IGs (red and ellipse). (**B**) GO terms of PRIGs: on the left is the gene, up-regulation was red and down-regulation was blue, on the right was a different GO term, and the genes were linked via ribbons to their assigned terms (*P*<0.05). (**C**) The TOP-10 terms of BP and MF. (**D**) GSEA analysis of the differentially expressed genes in the high-risk group. PRIGs, prognosis-related immune genes; PPI, Protein-Protein interaction;GO, Gene Ontology; BP, biological processes; MF, molecular functions;GSEA,Gene Set Enrichment Analysis.

The PPI networks between DIGs and DTFs were made up of ten DEIGs and four DETFs. All of them had a positive relationship (correlation coefficient = 0.4, *P*<0.001, Figure 4A). GO enrichment analysis of the 12 PRIGs manifested in Figure 4B,C, a total of 36 GO terms of BP, 26 GO terms of MF were identified to be significant (Supplementary Material S1, FDR < 0.05). These 12 genes were involved in several essential BPs, including hormone secretion, hormone transport, lipid transport, lipid localization, signal release, macrophage differentiation regulation, negative regulation of blood pressure, and negative regulation of secretion, glucose homeostasis, brown fat cell differentiation. The TOP-10 terms of BP and MF are shown in Figure 4C.

The KEGG pathway analysis showed that PRIGs are mainly enriched in the cytokine–cytokine receptor interaction, Peroxisome Proliferators-activated Receptor (PPAR) signaling pathway (*P*<0.05). GSEA analysis showed that changed genes were enriched in several common pathways. A total of 111/178 genesets were up-regulated in phenotype high-risk group, 66 gene sets were significant at FDR < 25%. The top-eight of the high-risk group are shown in Figure 4D, including hedgehog signaling pathway (Enrichment score, NES = 2.09, *P*=0.000), basal cell carcinoma (NES = 1.98, *P*=0.000), axon guidance (NES = 1.87, *P*=0.004), notch signaling pathway (NES = 1.86, *P*=0.010), Gonadotropin-releasing hormone (GnRH) signaling pathway (NES = 1.84, *P*=0.002), glycosaminoglycan biosynthesis chondroitin sulfate (NES = 1.83, *P*=0.006), extracellular matrix (ECM) receptor interaction (NES = 1.77, *P*=0.025), and dorsoventral axis formation (NES = 1.77, *P*=0.011).

## Discussion

**Figure 5:**
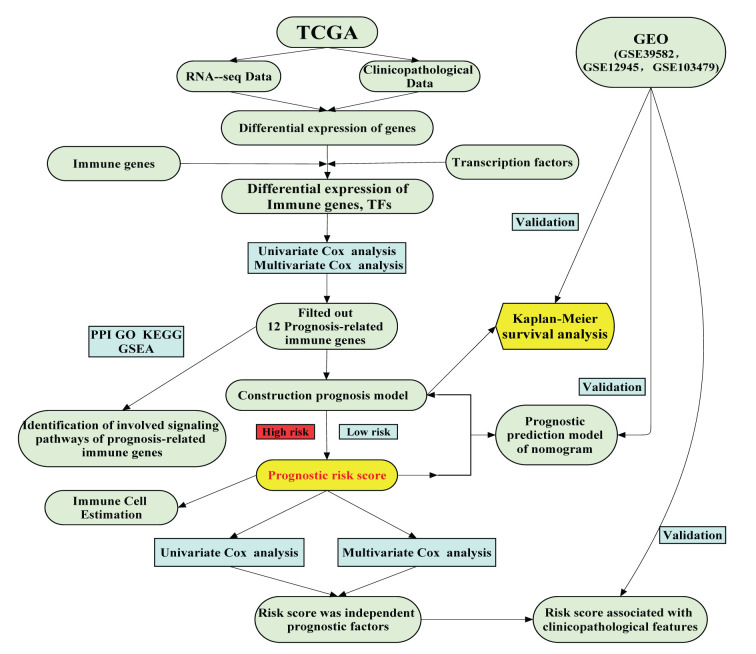
A flowchart of the study design and analysis

The diagnostic strategy for evaluating the tumor immune infiltration has a comprehensive clinical application value. Although the overlapping biological features of tumor immunophenotypes have been described [3], gene composition lacks consensus, the cell specificity of gene expression signal is not apparent, and there is no systematic analysis of the gene’s prognostic value in various tumor types. Until recently, a comprehensive meta-analysis confirmed the prognostic role of the IG in cancer as a whole [24]. In the current work, we singled out 24 PRIGs and constructed a prognosis model based on 12 PRIGs, which was entirely accurate and had better predictive value. A short bioinformatics protocol flow chart listed in Figure 5.

First, univariate regression analysis demonstrated that 22 genes had a relationship with poor prognosis, especially *SLC10A2, PLCG2*, and *PTH1R*. Previous reports have mentioned that secondary bile acids increase the risk of COAD, and *SLC10A2* plays a crucial role in intestinal bile acid reabsorption. This transporter is the primary mechanism for the uptake of intestinal bile acids by apical cells in the distal ileum. Bile acid malabsorption is related to epidermal growth factor receptor expression and M3 muscarinic [25,26]. PLCG2 is associated with immune responses and PLCG2 mutations have been found in the chronic lymphocytic leukemia patient [27,28]. However, its role in COAD’s occurrence and development is not very clear, and is worth further study. Researchers found that PTH1R was associated with both miRNA and DNA methylation in colorectal liver metastasis patients [29].

Second, whether the IG expression can be used as a prognostic marker is a vital research topic. In the present study, our prognosis model is considered to be valuable. The 1-, 3-, and 5-year AUC values of ROC were 0.792, 0.744, and 0.797, respectively, which was more accurate than previous reports [30,31]. The 12 IGs may be ideal prognostic markers. Five-year OS was ∼75% in the low-risk group and 50% in the high-risk group, consistent with the previous report [32]. High-risk genes in the model, including *SLC10A2, NOX4, FABP4, ADIPOQ, IGKV1-33, IGLV6-57, INHBA, UCN, VIP, NGFR*, and *TRDC*. NADPH oxidase complex can positively predict relapse in stage II left-side COAD, NOX4 encodes a member of the NOX-family of enzymes that functions as the catalytic subunit [33]. FABP4 plays a crucial role in regulating lipid metabolism and adipocyte differentiation associated with the tumor suppressor, PTEN [34]. FABP4 and ADIPOQ have been reported to increase the risk of colorectal neoplasia related to the COAD survival probability [35,36]. Dudgeon et al. found that UCN can inhibit COAD cell growth via inducing apoptosis by PUMA and a p53 target [37]. VIP promotes the initiation and progression of COAD by the generation of a local proinflammatory environment [38]. NGFR hypermethylation can be observed through COAD development [39]. The risk score is closely correlated with the COAD malignant clinicopathological characteristics and is an independent prognostic factor. The prediction ability of 12 immune genes is also cross-verified in three GEO data sets, which shows that our cohort analysis based on TCGA and GEO data has predictive value. Moreover, the roles of IGKV1-33, IGLV6-57, and TRDC in the development of COAD are not very clear, which deserve further study.

Subsequently, we constructed a nomogram to predict individual clinical outcomes. Nomogram is a stable and reliable tool to quantify individual risk by combining and describing risk factors, which has been used for tumor prognosis, including colorectal cancer [40,41]. By summarizing all points of view, the model provides individuals with numerical possibilities for clinical outcomes, such as OS, relapse, and drug noncompliance. Except for standard clinicopathological features, such as genetic markers, can also be included in predictive nomogram models to predict clinical outcomes [41,42]. The combination of autophagy gene features and prognostic factors has a better prognostic value than a single application [43]. Besides, the calibration curve showed that the nomogram accurately predicted 3- and 5-years survival rates. Consistently, in the current study, we illustrated that a nomogram, including the 12 PRIGs signature, could better predict the 3- and 5-years survival possibility of COAD patients. These results suggest that the 12-PRIGs prognostic model maybe have a convinced value in adjusting COAD patients’ treatment plans.

Furthermore, GO enrichment and KEGG pathway analysis show that PRIG participates in regulating several crucial processes and signaling pathways. ADIPOQ, INHBA, UCN, and VIP participate in hormone secretion and hormone transport BPs. SLC10A2, FABP4, ADIPOQ, and INHBA involved in lipid transport and lipid localization BPs [44–46]; INHBA, CXCL3, and NGFR participate in the regulation of the cytokine–cytokine receptor interaction pathway; FABP4 and ADIPOQ involved in the regulation of PPAR signaling pathway, which coincided with the previous report, PPAR signaling pathway might affect the recurrence of COAD patients [47,48]. The GSEA analysis further suggested that the pro-tumor inflammatory reaction might be more prominent in stage IIB/IIC of COAD [49]. The blockade of PD-L1 increased FABP4 expression in tissue-resident memory T (Trm) cells, promoting lipid uptake by Trm cells [50]. Previous reports indicate that the hedgehog signaling pathway might affect the recurrence of COAD patients [51]. Dandawate et al. reported that the Notch signaling pathway involved regulating colon cancer cells’ proliferation [51]. Our results are consistent with these reports.

From the above discussion, the conclusion can be reached that 12 IGs were more significantly associated with the COAD prognosis and the prognosis model was accurate. The prognostic model based on these genes is validated in three independent GEO cohorts. Further research might be necessary about these genes in the clinical application and may provide a new perspective into the COAD’s pathogenesis. Some of the antecedently overlooked genes maybe have a chance to become additional biomarkers for COAD. Finally, the present study enhanced our cognition of the sophistication of the occurrence and development of COAD and may detect novel therapeutic targets.

## Supplementary Material

Supplementary Figures S1-S3 and Tables S1-S2

Supplementary Material file

## Data Availability

The authors certify that all the original data in this research could be obtained from public database. Both the transcriptome and clinical data of COAD were obtained from the TCGA database (https://tcga-data.nci.nih.gov/tcga/). The data of training group are available at NCBI GEO (GSE39582 https://www.ncbi.nlm.nih.gov/geo/query/acc.cgi?acc=GSE39582, GSE12945 https://www.ncbi.nlm.nih.gov/geo/query/acc.cgi?acc=GSE12945, and GSE103479 https://www.ncbi.nlm.nih.gov/geo/query/acc.cgi?acc=GSE103479). Other data that were used to support the findings of the present study are included within the supplementary information files. All the raw data in the present study are available from the first author or corresponding author upon request.

## Abbreviations

AUC: Area Under Curve
BP: Biological Process
COAD: Colon Adenocarcinoma
C-index: Consistency Index
DAVID: The Database for Annotation, Visualization and Integrated Discovery
DEG: Differentially Expressed Gene
DEIG: Differentially Expressed Immune Gene
DETF: Differentially Expressed Transcription Factor
ECM: Extracellular Matrix
GEO: Gene Expression Omnibus
GnRH: Gonadotropin-releasing Hormone
GO: Gene Ontology
GSEA: Gene Set Enrichment Analysis
HR: Hazard Ratio
IG: Immune Gene
KEGG: Kyoto Encyclopedia of Genes and Genomes
MF: Molecular Function
NES: Enrichment Score
OS: Overall Survival
PPAR: Peroxisome Proliferators-activated Receptor
PPI: Protein–Protein Interaction
PRIG: Prognosis-related IGs
RMA: Robust Multi-Array Average
ROC: Receiver Operating Characteristic
TCGA: The Cancer Genome Atlas
TF: Transcription Factor
TME: Tumor Microenvironment
TrmR: Resident memory T cell
