# Supplementary material for: Identification prognosis-associated immune genes in colon adenocarcinoma
Source: Biosci Rep. 2020 Nov 17;40(11):BSR20201734. doi: 10.1042/BSR20201734 (PMC7670579; doi:10.1042/BSR20201734)
Supplement: Supplementary Figures S1-S3 and Tables S1-S2 [file BSR-2020-1734_supp.pdf]

	pvalue	Hazard ratio
SLC10A2	0.008	1.833(1.174−2.860)
CXCL3	0.007	0.976(0.959−0.993)
NOX4	0.009	1.603(1.126−2.283)
FABP4	<0.001	1.014(1.007−1.020)
ADIPOQ	<0.001	1.102(1.047−1.160)
CCL19	0.006	1.030(1.008−1.051)
PLCG2	0.003	1.676(1.192−2.355)
IGHG1	<0.001	1.001(1.000−1.001)
IGHG4	0.008	1.000(1.000−1.001)
IGHV4−31	0.008	1.008(1.002−1.014)
IGHV5−51	0.002	1.002(1.001−1.003)
IGKV1−33	0.002	1.030(1.011−1.050)
IGKV1−8	0.002	1.044(1.015−1.073)
IGLV6−57	0.003	1.002(1.001−1.003)
SEMA3G	<0.001	1.294(1.124−1.491)
INHBA	<0.001	1.054(1.022−1.086)
STC1	0.007	1.078(1.020−1.139)
UCN	0.003	1.386(1.120−1.715)
VIP	0.002	1.058(1.021−1.096)
NGFR	<0.001	1.201(1.092−1.320)
NPR1	0.004	1.504(1.136−1.991)
OXTR	<0.001	1.421(1.171−1.724)
PTH1R	<0.001	1.628(1.220−2.173)
TRDC	0.009	1.148(1.035−1.273)

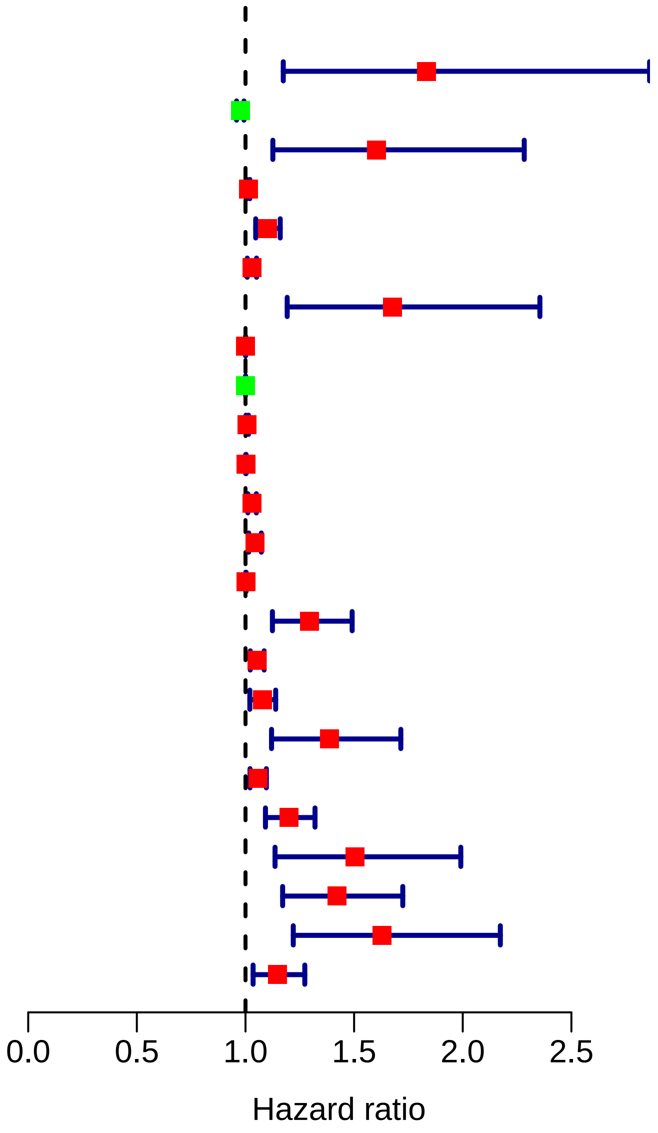

Figure S1. The hazard ratios (HR), 95% confidence intervals (CI) calculated by univariate Cox regression.

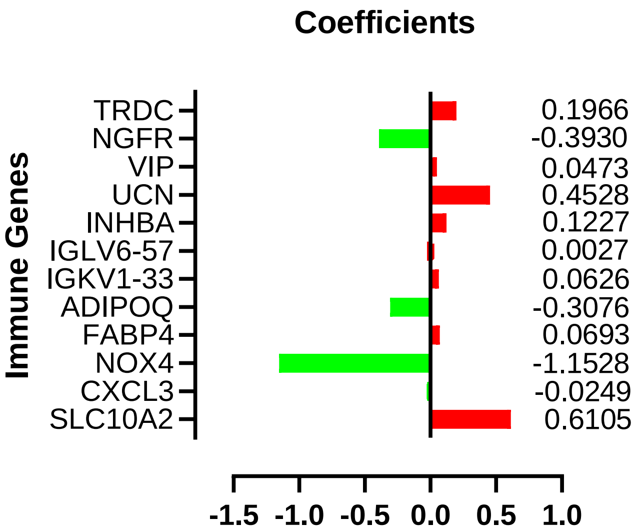

Coefficients
Immune Genes
TRDC
NGFR
VIP
UCN
INHBA
IGLV6-57
IGKV1-33
ADIPOQ
FABP4
NOX4
CXCL3
SLC10A2
0.1966
-0.3930
0.0473
0.4528
0.1227
0.0027
0.0626
-0.3076
0.0693
-1.1528
-0.0249
0.6105
-1.5
-1.0
-0.5
0.0
0.5
1.0

Figure S2. The coefficient of PRIGs.

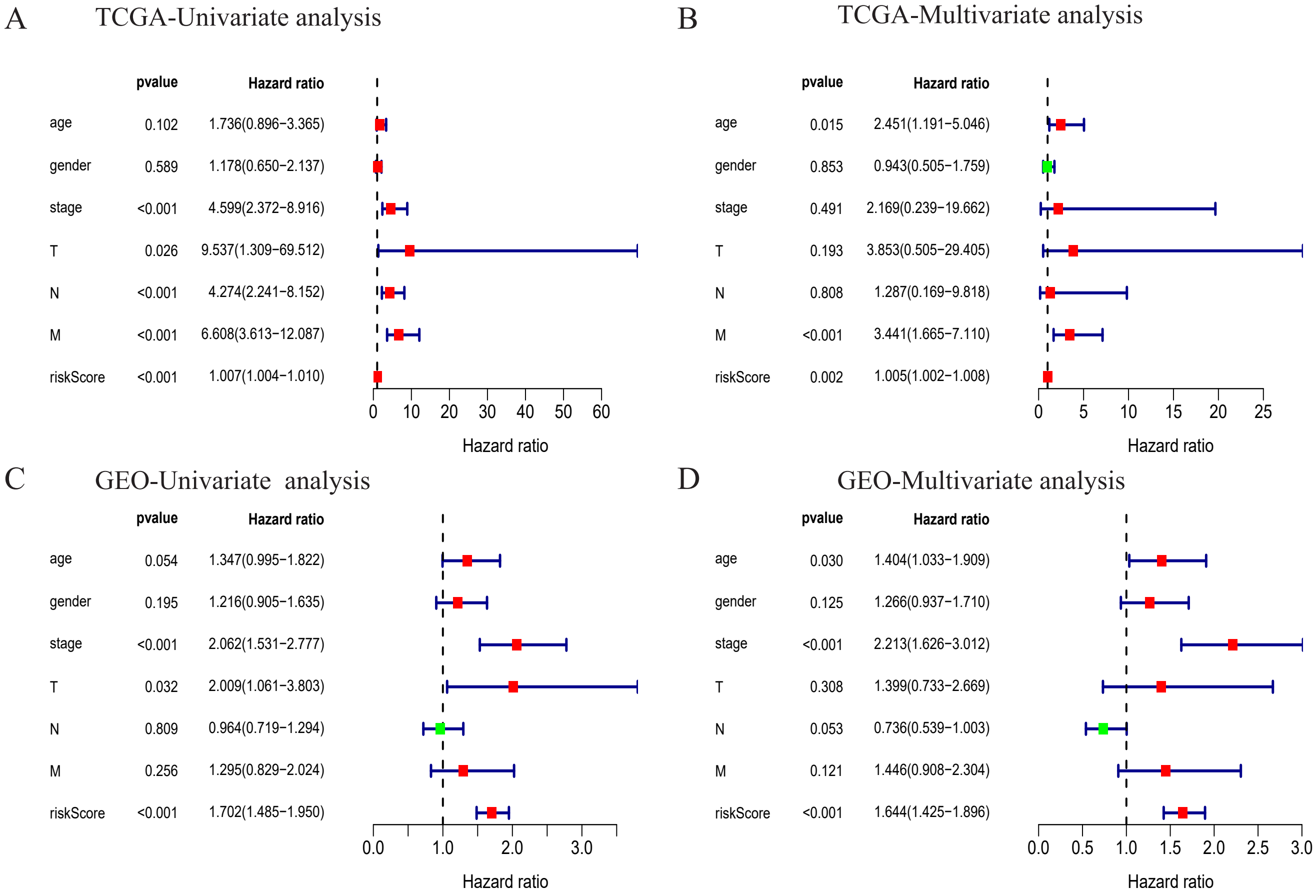
A
TCGA-Univariate analysis
pvalue
Hazard ratio
age 0.102 1.736(0.896−3.365)
gender 0.589 1.178(0.650−2.137)
stage <0.001 4.599(2.372−8.916)
T 0.026 9.537(1.309−69.512)
N <0.001 4.274(2.241−8.152)
M <0.001 6.608(3.613−12.087)
riskScore <0.001 1.007(1.004−1.010)
0 10 20 30 40 50 60
Hazard ratio
B
TCGA-Multivariate analysis
pvalue
Hazard ratio
age 0.015 2.451(1.191−5.046)
gender 0.853 0.943(0.505−1.759)
stage 0.491 2.169(0.239−19.662)
T 0.193 3.853(0.505−29.405)
N 0.808 1.287(0.169−9.818)
M <0.001 3.441(1.665−7.110)
riskScore 0.002 1.005(1.002−1.008)
0 5 10 15 20 25
Hazard ratio
C
GEO-Univariate analysis
pvalue
Hazard ratio
age 0.054 1.347(0.995−1.822)
gender 0.195 1.216(0.905−1.635)
stage <0.001 2.062(1.531−2.777)
T 0.032 2.009(1.061−3.803)
N 0.809 0.964(0.719−1.294)
M 0.256 1.295(0.829−2.024)
riskScore <0.001 1.702(1.485−1.950)
0.0 1.0 2.0 3.0
Hazard ratio
D
GEO-Multivariate analysis
pvalue
Hazard ratio
age 0.030 1.404(1.033−1.909)
gender 0.125 1.266(0.937−1.710)
stage <0.001 2.213(1.626−3.012)
T 0.308 1.399(0.733−2.669)
N 0.053 0.736(0.539−1.003)
M 0.121 1.446(0.908−2.304)
riskScore <0.001 1.644(1.425−1.896)
0.0 0.5 1.0 1.5 2.0 2.5 3.0
Hazard ratio

Figure S3. Prognosis model of immune genes. **(A, C)** Univariate Cox regression analysis. Forest plot of the association between risk factors and survival of TCGA-COAD **(A)** and GEO-COAD **(C).(B, D)** Multivariate Cox regression analysis. The risk score was an independent prognostic element in TCGA-COAD **(B)** and GEO-COAD **(D).**

Table S1. Characteristics of training and test group.

		TCGA (Training group)	GEO (Test group)
**Age(years)**		67.01±12.77	66.21±13.23
**Gender**	Female	180	262
	Male	205	325
**Stage**	Stage Ⅰ	66	45
	Stage Ⅱ	151	271
	Stage Ⅲ	103	212
	Stage Ⅳ	54	56
	Unknow	11	3
**T (Tumor)**	T1	10	11
	T2	68	57
	T3	263	388
	T4	44	108
	Tis	1	3
	Unknow	0	20
**N (Lymph Node)**	N0	231	317
	N1	88	141
	N2	66	99
	N3	0	5
	NX	0	25
**M (Metastasis)**	M0	286	508
	M1	54	57
	Mx	40	3
	Unknow	5	19

Table S2.Prognosis-related immune genes (univariate Cox regression analysis)

PRIGs	HR	HR.95L	HR.95H	*P* value
CXCL3	0.976274178	0.959433832	0.993410111	0.006836376
IGHG4	1.000462714	1.000118581	1.000806966	0.008401804
IGHG1	1.000557152	1.000229499	1.000884911	0.000858607
IGHV5-51	1.00161858	1.00058123	1.002657006	0.00222059
IGLV6-57	1.00196457	1.000646266	1.003284611	0.003480915
IGHV4-31	1.008180525	1.002111576	1.014286228	0.008176801
FABP4	1.013514986	1.006982636	1.020089712	4.72E-05
CCL19	1.029586494	1.008468596	1.051146613	0.005824639
IGKV1-33	1.030336759	1.011010023	1.05003295	0.001979212
IGKV1-8	1.043893862	1.015268705	1.073326095	0.002460688
INHBA	1.053507046	1.022173509	1.085801076	0.000715416
VIP	1.057597466	1.020679968	1.095850251	0.002007728
STC1	1.077990496	1.020432945	1.138794584	0.007308441
ADIPOQ	1.101937111	1.046564847	1.160239044	0.000224086
TRDC	1.148292064	1.035438282	1.273445928	0.008799448
NGFR	1.200503367	1.092020089	1.319763572	0.000155797
SEMA3G	1.294274954	1.123577659	1.490905097	0.000350682
UCN	1.386191418	1.120402289	1.715032777	0.002640879
OXTR	1.420766913	1.170702262	1.72424594	0.000377164
NPR1	1.504102806	1.13612177	1.991270048	0.004351938
NOX4	1.603287544	1.126141305	2.282600714	0.008816796
PTH1R	1.628067123	1.219526757	2.173468145	0.000945574
PLCG2	1.675520381	1.192276181	2.354629398	0.002949259
SLC10A2	1.832753743	1.174436133	2.860084245	0.007628351
